# Previous exposure to musical auditory stimulation immediately influences the cardiac autonomic responses to the postural change maneuver in women

**DOI:** 10.1186/1755-7682-6-32

**Published:** 2013-08-14

**Authors:** Bianca CR de Castro, Heraldo L Guida, Adriano L Roque, Luiz Carlos de Abreu, Lucas L Ferreira, Rodrigo D Raimundo, Carlos BM Monteiro, Flávia C Goulart, Celso Ferreira, Renata S Marcomini, Vivian F Ribeiro, Alessandro HN Ré, Luiz Carlos M Vanderlei, Vitor E Valenti

**Affiliations:** 1 Department of Speech Language and Hearing Therapy, Faculty of Philosophy and Sciences, UNESP, Av. Hygino Muzzi Filho 737. 17.525-900 Marilia, SP, Brazil; 2 Department of Medicine, Cardiology Division, UNIFESP, Rua Sena Madureira 1500 04021-001 Sao Paulo, SP, Brazil; 3 Department of Morphology and Physiology, School of Medicine of ABC. Av. Principe de Gales, 821. 09060-650 Santo Andre, SP, Brazil; 4 School of Arts, Sciences and Humanities, University of São Paulo, USP. Av. Arlindo Béttio, 1000. 03828-000 Sao Paulo, SP, Brazil; 5 Department of Physiotherapy, Faculty of Sciences and Technology, UNESP, Rua Roberto Simonsen, 305. 19060-900 Presidente Prudente, SP, Brasil

**Keywords:** Autonomic nervous system, Auditory stimulation, Cardiovascular system, Music

## Abstract

**Background:**

Chronic exposure to musical auditory stimulation has been reported to improve cardiac autonomic regulation. However, it is not clear if music acutely influences it in response to autonomic tests. We evaluated the acute effects of music on heart rate variability (HRV) responses to the postural change maneuver (PCM) in women.

**Method:**

We evaluated 12 healthy women between 18 and 28 years old and HRV was analyzed in the time (SDNN, RMSSD, NN50 and pNN50) and frequency (LF, HF and LF/HF ratio) domains. In the control protocol, the women remained at seated rest for 10 minutes and quickly stood up within three seconds and remained standing still for 15 minutes. In the music protocol, the women remained at seated rest for 10 minutes, were exposed to music for 10 minutes and quickly stood up within three seconds and remained standing still for 15 minutes. HRV was recorded at the following time: rest, music (music protocol) 0–5, 5–10 and 10–15 min during standing.

**Results:**

In the control protocol the SDNN, RMSSD and pNN50 indexes were reduced at 10–15 minutes after the volunteers stood up, while the LF (nu) index was increased at the same moment compared to seated rest. In the protocol with music, the indexes were not different from control but the RMSSD, pNN50 and LF (nu) were different from the music period.

**Conclusion:**

Musical auditory stimulation attenuates the cardiac autonomic responses to the PCM.

## Background

Music therapy is considered as a health profession, as it consists of an interpersonal process in which a trained music therapist uses music and all of its sides—physical, social, emotional, mental, and spiritual—to help clients to improve or maintain their health. Music therapists mainly help people to develop their wellbeing across many areas (e.g., cognitive functioning, motor skills, emotional and affective development, behavior and social skills, and quality of life) by using music experiences (e.g., free improvisation, singing, songwriting, listening to and discussing music, moving to music) to achieve treatment goals and objectives [1]. Musical auditory stimulation has received attention in the literature [2-4].

Classical music stimulation presented beneficial effects on the cardiovascular system [5]. A previous study [5] investigated the effect of music with vocals, orchestra and progressive crescendos on middle cerebral artery flow, respiratory heart rate and blood pressure. Decreased sympathetic activity on the cardiovascular system was reported, suggesting involvement of the autonomic nervous system (ANS) on the heart.

The ANS is able to provide information regarding the condition of the organism [6]. A non-invasive method for investigation of the ANS that describes the oscillations of the intervals between consecutive heartbeats is the heart rate variability (HRV). This method is a conventionally accepted term to describe the fluctuations in the intervals between consecutive heartbeats (RR intervals), which are indicated to influence the sinusal node [7].

Examination of autonomic cardiovascular tests is indicated to offer relevant information regarding adequate function of the ANS modulation on the heart. It is used for cardiovascular system control investigation in healthy subjects, in adult patients with different diseases and for the diagnosis of autonomic dysfunction. One common cardiovascular test used in the clinical routine is the postural change maneuver (PCM), a test based on the measurement of heart rate reflex changes in response to postural change stimulation [8].

Our group has previously reported that classical baroque music style acutely influences HRV [9,10]. However, there are a lack of investigations in the literature that report the influence of musical auditory stimulation on autonomic cardiac responses to autonomic tests. In addition, the PCM is a simple, non-invasive and non-expensive autonomic test used in clinical routine. It is hypothesized that previous exposure to music influences the cardiac autonomic regulation responses to autonomic tests. Therefore, we endeavored to evaluate the acute effects of classical musical auditory stimulation on HRV responses to PCM in women.

## Method

### Study population

We analyzed 12 healthy female subjects between 18 and 28 years old, selected from our institution. All volunteers were informed about the procedures and objectives of the study and, after agreeing, signed a term of informed consent. All procedures were approved by the Ethics Committee in Research of the Faculty of Sciences of the Universidade Estadual Paulista, Campus of Marilia (Protocol No. CEP-2011-382) and followed the resolution 196/96 National Health 10/10/1996.

### Exclusion criteria

We considered the following exclusion criteria: auditory and cardiopulmonary disorders, neurological and other impairments that prevent the subject to perform the protocol such as orthostatic hypotension, orthopedic disorders and disability to perform the procedures. Exclusion criteria also included subjects under treatment with drugs that influence cardiac autonomic regulation, i.e. beta-blockers, antidepressive agents, beta-adrenergic agonist receptors, dopaminergic agonist receptors. We also excluded subjects with previous experience with musical instruments and classic ballet dance, since it affects cardiovascular responses [11].

### Initial evaluation

Before the experimental procedure, volunteers were identified by collecting the following information: age, gender, weight, height and body mass index (BMI). Anthropometric measurements were obtained according to Lohman et al. [12]. Body mass index (BMI) was calculated using the following formula: weight (kg)/height (m)^2^.

### Experimental protocol

Data were collected in our laboratory under controlled temperature (21°C–25°C) and humidity (50%–60%), and volunteers were instructed to avoid consuming alcohol, caffeine and substances that influence the ANS for 24 hours before evaluation. Data were collected between 8 a.m. and 12 a.m. All procedures necessary for the data collection were explained to the individuals, and the subjects were instructed to remain at rest and not to talk during the data collection.

After the initial evaluation, the heart monitor strap was placed on each subject’s thorax over the distal third of the sternum. A HR receiver (Polar RS800CX monitor, Polar Electro OY, Kempele, Finland), responsible for collecting the HR and R-R intervals, was placed on the wrist.

In the control protocol, the subject remained at seated rest for 10 minutes with spontaneous breathing. After ten minutes, the volunteers quickly stood up from a seated position within three seconds according to verbal command and remained standing for 15 minutes. In the music protocol, the subject remained at seated rest for 10 minutes with spontaneous breathing. Subsequently, the subjects were exposed to a classical musical auditory stimulation (Pachelbel-Canon in D) for an additional 10 minutes. After the music exposure, the volunteers quickly stood up from a seated position within three seconds according to verbal command and remained standing for 15 minutes. The sequence of the protocols was randomized based on coded opaque envelopes.

In order to further investigate the effects of music on cardiac autonomic regulation, we performed an additional protocol to compare the HRV indices between standing position with no music exposure and standing position with music exposure.

### HRV analysis

The R-R intervals recorded by the portable HR monitor (with a sampling rate of 1000 Hz) were uploaded to the Polar Precision Performance program (v. 3.0, Polar Electro, Finland). The software enabled the visualization of HR and the extraction of a cardiac period (R-R interval) file in downloadable “.txt” format. Following digital filtering complemented with manual filtering for the elimination of premature ectopic beats and artifacts, at least 256 R–R intervals were used for the data analysis. Only series with more than 95% sinus rhythm was included in the study. HRV was analyzed at four moments: seated rest with spontaneous breathing, 10 minutes of music exposure (in the protocol with music exposure), 0–5 minutes, 5–10 minutes and 10–15 minutes after the subjects stood up. We evaluated the linear indices of HRV. For calculation of the indices we used the HRV Analysis software (Kubios HRV v.1.1 for Windows, Biomedical Signal Analysis Group, Department of Applied Physics, University of Kuopio, Finland) [13].

### Linear indices of HRV

To analyze HRV in the frequency domain, the low frequency (LF =0.04 to 0.15 Hz) and high frequency (HF = 0.15 to 0.40 Hz) spectral components were used in normalized units (nu), which represents a value relative to each spectral component in relation to the total power minus the very low frequency (VLF) components, and the ratio between these components (LF/HF). The spectral analysis was calculated using the Fast Fourier Transform algorithm [14,15].

The analysis in the time domain was performed by means of SDNN (standard deviation of normal-to-normal R-R intervals), the percentage of adjacent RR intervals with a difference of duration greater than 50 ms (pNN50) and RMSSD (root-mean square of differences between adjacent normal RR intervals in a time interval) [14].

### Statistical analysis

Standard statistical methods were used for the calculation of means and standard deviations. Normal Gaussian distribution of the data was verified by the Shapiro-Wilk goodness-of-fit test (z value >1.0). Regarding the first protocol for parametric distributions, we applied the ANOVA for repeated measures followed by the Bonferroni post-test (SDNN) and for non-parametric distributions we used the Friedman test followed by the Dunn’s test (1st protocol: RMSSD, pNN50, LF [nu], HF [nu] and LF/HF). We compared the HRV indices between the four moments in the first protocol (seated rest vs. 0–5 min after the volunteers stood up vs. 5–10 min after the volunteers stood up vs. 10–15 min after the volunteers stood up). In relation to the second protocol for parametric distributions, we applied the ANOVA for repeated measures followed by the Bonferroni post-test (SDNN), for non-parametric distributions we used the Friedman test followed by the Dunn’s test (RMSSD, pNN50, LF [nu], HF [nu] and LF/HF). We compared the HRV indices between the five moments in the second protocol (seated rest vs. 10 minutes musical auditory stimulation vs. 0–5 min after the volunteers stood up vs. 5–10 min after the volunteers stood up vs. 10–15 min after the volunteers stood up). Differences were considered significant when the probability of a Type I error was less than 5% (*p* < 0.05). We used the Software GraphPad StatMate version 2.00 for Windows, GraphPad Software, San Diego California USA.

## Results

**Figure 1:**
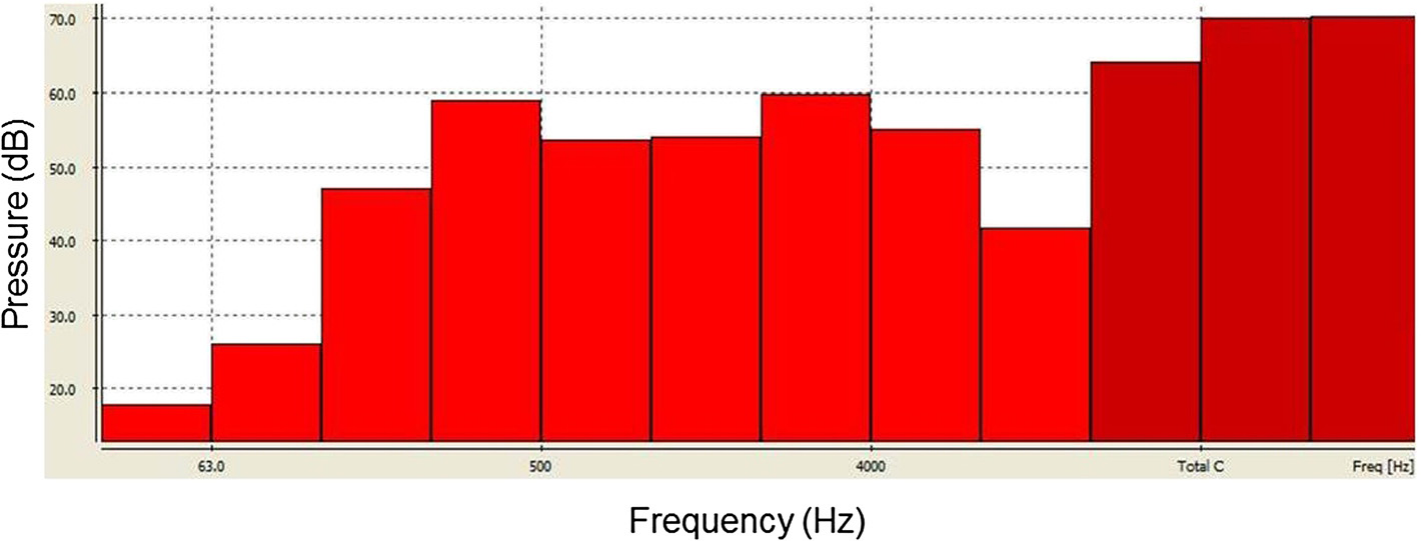
**Equivalent sound level of auditory musical stimulation of classical style.** dB: Decibel; Hz: hertz.

**Figure 2:**
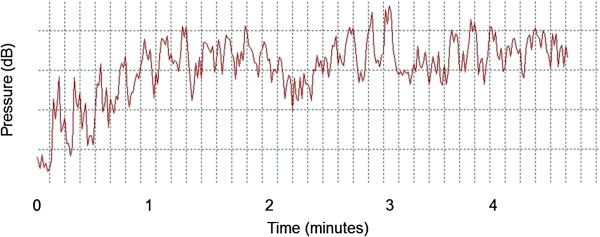
**Frequency sound level of auditory musical stimulation of classical style.** dB: Decibel.

The volunteers were exposed to an equivalent sound level between 65 and 80 dB. Figure 1 and Figure 2 show the equivalent and frequency sound level during classical music auditory stimulation, respectively.

Data for baseline systolic (SAP) and diastolic arterial pressure (DAP), heart rate (HR) and mean RR interval, age, height, body weight and body mass index (BMI) are presented in Table 1.

**Table 1:** Baseline diastolic (DAP) and systolic arterial pressure (SAP), heart rate (HR), mean RR interval, weight, height and body mass index (BMI) of the volunteers

**Variable**	**Value**
Height (m)	1.68±0.07
Weight (kg)	54.4±35
BMI (kg/m^2^)	23.9±4
HR (bpm)	80.9±9
Mean RR (ms)	80.9±9
SAP (mmHg)	104.8±12
DAP (mmHg)	70.5±10

Mean±standard deviation. N=12002E.

Legend: *m* meters, *ms* millisecond, *kg* kilograms, *bpm* beats per minute, *mmHg* millimeters of mercury.

Table 2 presents data related to the time domain indices at seated and after the volunteers stood up. We noted that the SDNN, RMSSD and pNN50 indices were reduced between 10 and 15 minutes after the subjects stood up compared to seated rest in the control protocol (control vs. 10–15 min).

**Table 2:** Mean and standard deviation for time-domain indices between before, 0–5 minutes, 5–10 minutes and 10–15 minutes after the postural change maneuver in the control protocol

**Index**	**Seated rest**	**0-5 min**	**5-10 min**	**10-15 min**	**p**
SDNN (ms)	45.1±9	44.6±13	44.6±13	*34.7±8	0.040
RMSSD (ms)	30.4±12	19.2±7	17.7±7	*16.9±7	0.030
pNN50 (%)	11.4±14	3.2±4	2.7±3	*2±3	0.0189

*Different from Control. N=12.

Legend: *SDNN* standard deviation of normal-to-normal R-R intervals, *pNN50* the percentage of adjacent RR intervals with a difference of duration greater than 50 ms, *RMSSD* root-mean square of differences between adjacent normal RR intervals in a time interval. *ms* millisecond.

Table 3 displays results concerning the frequency domain indices at seated and after the subjects stood up in the control protocol. We observed that the LF (nu) index increased at 10–15 minutes after the subjects stood up compared to control (seated vs. 10–15 min). The HF (nu) index tended to be reduced and the LF/HF ration tended to increase compared to control.

**Table 3:** Mean and standard deviation for time-domain indices between before and after the postural change maneuver in the control protocol

**Index**	**Seated rest**	**0-5 min**	**5-10 min**	**10-15 min**	**p**
LF (nu)	63±18	73.4±19	74.7±19	*74.3±18	0.020
HF (nu)	30±18	26.6±19	20.3±12	25.7±18	0.060
LF/HF	2.7±2	4.7±3	5±3	5±3	0.062

*Different from Control. N=12.

Legend: *LF* low frequency, *HF* high frequency, *LF/HF* - low frequency/high frequency ration, *ms* milliseconds, *nu* normalized units.

Table 4 displays data of the time domain indices at seated position and after the subjects stood up in the protocol exposed to musical auditory stimulation. The indexes were not different compared to the control seated rest. The RMSSD and the pNN50 indices were reduced at 5–10 minutes compared to the period during music exposure. Nonetheless, the SDNN index was not changed.

**Table 4:** Mean and standard deviation for time-domain indices between before and after the postural change maneuver in the music protocol

**Index**	**Seated rest**	**Music**	**0-5 min**	**5-10 min**	**10-15 min**	**p**
SDNN (ms)	44.2±14	53.7±16	50.2±14	47.6±11	46.2±9	0.580
RMSSD (ms)	35.7±15	37.4±15	24.9±9	*21.7±7	22.5±6	0.023
pNN50 (%)	16.9±16	17.7±15	7.2±7	*4.1±4	4.6±4	0.030

*Different from Music. N=12.

Legend: *SDNN* standard deviation of normal-to-normal R-R intervals, *pNN50* the percentage of adjacent RR intervals with a difference of duration greater than 50 ms, *RMSSD* root-mean square of differences between adjacent normal RR intervals in a time interval, *ms* millisecond.

In Table 5, we present data regarding the frequency domain indices at seated position and after volunteers stood up in the same protocol exposed to musical auditory stimulation. The indexes did not change compared to the control seated rest. We revealed that the LF (nu) index increased at 0–15 minutes compared to the period during music exposure, while the LF/HF ratio increased at 0–5 minutes after the women stood up compared to the period during music exposure. The HF (nu) index reduced 0–5 minutes compared to the period during music exposure.

**Table 5:** Mean and standard-deviation for time-domain indices between before and after the orthostatic test in the music protocol

**Index**	**Seated rest**	**Music**	**0-5 min**	**5-10 min**	**10-15 min**	**p**
LF (nu)	67.5±19	63.5±19	*80±10	*79.7±10	*79.9±10	0.001
HF (nu)	32.5±19	36.5±19	*20±10	20.3±10	20.1±10	0.010
LF/HF	4.3±5	2.4±1	*6±5	5.9±5	5.7±4	0.030

*Different from Music. N=12.

Legend: *LF* low frequency, *HF* high frequency, *LF/HF* low frequency/high frequency ration. *ms* milliseconds, *nu* normalized units.

In Table 6 and Table 7, we observe that musical auditory stimulation at standing position did not affect the time and frequency domain indices of HRV in the volunteers.

**Table 6:** Mean and standard deviation for time-domain indices between before and after the musical auditory stimulation at seated position

**Index**	**Standing**	**Music**	**p**
SDNN (ms)	43.8±9	37.4±11	0.343
RMSSD (ms)	38.1±17	30.8±14	0.224
pNN50 (%)	17.4±16	12.5±13	0.402

N=12.

Legend: *SDNN* standard deviation of normal-to-normal R-R intervals, *pNN50* - the percentage of adjacent RR intervals with a difference of duration greater than 50 ms, *RMSSD* root-mean square of differences between adjacent normal RR intervals in a time interval. *ms* millisecond.

**Table 7:** Mean and standard-deviation for time-domain indices between between before and after the musical auditory stimulation at seated position

**Index**	**Standing**	**Music**	**p**
LF (nu)	61.2±18	61±19	0.486
HF (nu)	36.9±18	38.7±19	0.341
LF/HF	2.6±2	2.3±1	0.171

N=12.

Legend: *LF* low frequency, *HF* high frequency, *LF/HF* low frequency/high frequency ration. *ms* milliseconds, *nu* normalized units.

## Discussion

Our study aimed to investigate the effects of previous musical auditory stimulation on cardiac autonomic responses to the PCM in healthy women. The expected response of the autonomic modulation of the heart to this test is an initial parasympathetic reduction and delayed increase of sympathetic modulation on the heart [8]. As a main finding, musical auditory stimulation attenuated the responses induced by the PCM, since the HRV indices were not different from the seated position in the music protocol. In the control protocol, all time domain indices were changed at 10–15 minutes after the change from seated to standing compared to the seated rest. On the other hand, when the subjects were exposed to musical auditory stimulation, we found no differences between the seated position (with no music) and the moments after the volunteers stood up. The cardiac autonomic responses in the standing position were compared to the sitting condition applied immediately after the music exposure. Moreover, in order to further investigate the effects of music on cardiac autonomic regulation at standing position we compared the HRV indices between standing position with no music exposure and standing position with music exposure. As no difference between the two moments was reported, it supports that previous exposure to the music used in our study influences the cardiac autonomic responses to the PCM without influence basal HRV.

We observed in the music protocol no change in global HRV (SDNN), a clear decrease of the parasympathetic component and an increase of the sympathetic tone (LF nu) on the heart compared to the music exposure, but not different from the seated position without music. To better understand the influence of musical auditory stimulation on cardiac autonomic response of PCM test, the effects of music at basal HRV during standing position were evaluated. We reported no significant difference between no music and during musical auditory stimulation moments at standing position, indicating that this style of music does not influence the basal HRV. It is suggested that although this music style caused no significant change in basal HRV it affects the HRV responses to the PCM.

Our results reported decreased values of the SDNN index at 10–15 minutes after the volunteers stoop up compared to the rest baseline in the control protocol. When the subjects were exposed to musical auditory stimulation before the PCM, there was no significant changed in the SDNN. This time domain index corresponds to the both parasympathetic and sympathetic activity. It is known to correspond to the global autonomic regulation of the heart. Nevertheless, it is not possible to distinguish whether changes in HRV are caused by increased sympathetic tone or withdrawal of vagal tone [16]. A recent study showed that chronic relaxant musical auditory stimulation increased the SDNN index in patients with breast cancer treated with anthracycline by the end of the 8-week treatment period [17], indicating improvement of global HRV [18]. However, the authors did not specify the equivalent sound level. In our study the volunteers were exposed to a musical auditory stimulation that ranged from 65 to 80 dB approximately. Our data suggest that previous acute exposure to the classical music used in our study attenuates the response of the SDNN index to the PCM.

In the control protocol, the LF frequency domain index was changed at 10–15 minutes after the volunteers stood up compared to the seated position. In the music protocol there was no change in the LF index. The LF index corresponds to the joint action of the vagal and sympathetic components on the heart, with a predominance of the sympathetic tone [16]. Normalizing data of the spectral analysis is used to minimize the effects of changes in the very low frequency (VLF) band. This is determined by dividing the power of a given component (LF or HF) by the total power spectrum, minus the VLF component and multiplied by 100. The VLF is less used in the literature, since its physiological significance is not well understood and seems to be related to the renin-angiotensin-aldosterone system, thermoregulation and the peripheral vasomotor tone [7,14]. Our finding suggests that the LF index response to the PCM is attenuated when the subject is exposed to musical auditory stimulation. However, based on this index and in the SDNN index, it is not clear whether the sympathetic or parasympathetic modulation is involved.

In this context, the responses of the RMSSD and pNN50 time domain indices to the PCM helped us to clarify which component of the cardiac autonomic regulation is involved. In the control protocol, the both indices were reduced at 10–15 minutes after the subjects stood up compared to the seated condition. In the music protocol, the RMSSD and the pNN50 indices were not changed compared to the seated position, indicating that the music influenced its responses to the PCM. The presence of harmonic and rhythmic sound may relieve pain and cause physical and emotional act on hemodynamic parameters such as heart rate, arterial blood pressure, temperature and relaxation in patients with regularization respiratory rate, muscle relaxation and improved sleep [19]. This mechanism is hypothesized to be involved in the attenuated responses of the parasympathetic tone on the heart to the PCM in subjects previously exposed to music.

In line with the parasympathetic components of the time domain indices, the frequency domain indices in response to the PCM were also influenced by the musical auditory stimulation. The HF index was not different at standing compared to the seated position in the both protocols, although it tended to decrease at standing compared to seated position in the control protocol (*p*=0.06). Some studies regarding musical auditory stimulation, medicine and psychology, described the anxiolytic responses caused by music. Those effects were evaluated for different music styles and for self-selected against experimenter-selected music. Perceived relaxation was elicited by sedative music, which is characterized as melodious, delicate, harmonic, and romantic [20], and by self-selected music [21]. The effects of musical auditory stimulation on physiological responses are not yet consistent. Previous investigations indicated that arterial blood pressure and heart rate were reduced in response to a sedative music style [22,23] and by self-selected music [24,25]. Other researches indicated that musical auditory stimulation presented no alterations on blood pressure or heart rate [26] and that any type of music increased physiological responses [20]. We believe that this inconsistency is due to the different tempos and characteristics of the all-classical music. Our study is the first to demonstrate an effect of musical auditory stimulation from Pachelbel (Canon in D) on the HRV in response to change of seated to standing in women.

HRV is a non-invasive method that evaluates the cardiac autonomic regulation. Because only HR is usually used as an index of anxiety and stress, changes in HR are supposed to be observed if music indeed decreases stress [27]. HRV is able to detect distinguished responses that simple changes in HR do not reveal. A previous study investigated the effects of music mainly by measuring average changes in heart rate from a baseline. Stress and anxiety influence not only reaction levels but also time series changes or variability in responses [28]. The findings in HRV reported in our study provide responses with a better specification compared to only heart rate measurement.

In our study, we used the composition from Pachelbel, Canon in D. The literature hypothesized that the inconsistent results with respect to heart rate alterations in response to music mentioned above are caused by individual response specificity in the ANS [29-31]. Our group intends to standardize a style of music in order to investigate the physiological responses induced by this musical auditory stimulation focusing on the cardiac autonomic regulation. To the best of our knowledge, no previous study already evaluated the effects of Pachelbel - Canon in D on the HRV.

According to our study, the subjects were exposed to music above 60 dB. It is know that white noise exposure above 50 dB increases sympathetic activity [18,32]. It was also reported high association between LF/HF ratio and noise intensity. Noise intensity was indicated to influence cardiac autonomic regulation. The mechanism involved in the cardiac autonomic responses to musical auditory stimulation occurs through several pathways [18]. An example is the startle reaction regulated the brainstem circuit. The acoustic startle reflex, a well-known effect of loud sounds on the cardiovascular physiology, is cited as a sudden traction of the arterial blood pressure and heart rate to an abrupt loud sound stimulation. The intensity used to elicit a startle reflex is approximately 110 dB, and the intensity is much louder than the music used in our study. On the other hand, the cardiovascular responses that habituated over trials were observed in the subjects evoked by repeated 60 dB and 110 dB white-noise stimulation [33]. The traction was considered as defense and startle response in humans or a fight/flight reaction in animals. The rise of cardiovascular parameters to acoustic startle stimulation suggested an autonomic function responding to the acoustic stimulation [34]. Moreover, cortical centers and also subcortical processing areas were supposed to be involved in the cardiovascular and hormonal responses to long-term stress activation by the environmental noises even though the noise intensity was as low as 53 dB [35].

Our study presents some important points to be raised. Since only healthy female subjects were evaluated to homogenize the study population, we should be careful when extrapolating this data to different gender and pathological conditions. Autonomic imbalance consisting of high sympathetic tone and decreased vagal modulation of the heart is implicated in the development of arrhythmic disorders and sudden cardiac death [7,15], our study is important to provide a new element, i.e. music, able to attenuate the cardiac autonomic responses to a stimulus that overcharge the heart and, as a consequence, reducing the heart demand. We found no significant (*p*>0.05) effect of music on basal HRV, this is possibly because the music used in this study was not entire relaxant and present instability between high and low tempos.

## Conclusion

Our findings indicated that previous musical auditory stimulation reduced the responses of the parasympathetic modulation of the heart to the PCM. Therefore, we suggest that this style of stimulation attenuated the cardiac autonomic responses to this test in women.

## Competing interest

The authors declare that they have no competing interest.

## Authors’ contribution

BCRC, HLG, ALR, LCA and VEV participated in the acquisition of data and revision of the manuscript. LLF, LCMV, CBMM, RDM, RSM and VEV conceived the study, determined the design, interpreted the data and drafted the manuscript. CF, LLF, RDR, RSM, VFR, CBMM¸ AHNR and DFS interpreted the data and drafted the manuscript. All authors read and gave final approval for the version submitted for publication.
